# Challenges of parents of young adults misusing psychoactive substances in small-scale mining communities in Ghana

**DOI:** 10.1080/28324765.2024.2382182

**Published:** 2024-07-26

**Authors:** Philip Asamoah, Samuel Nana Abokyi, Victor Fannam Nunfam

**Affiliations:** a Department of Social Work, School of Social Sciences, University of Ghana, Accra, Ghana; b History Department, Faculty of Social Sciences, University for Development Studies, Tamale, Ghana; c Social Development Section, Centre for Languages and Liberal Studies, Takoradi Technical University, Takoradi, Ghana; d School of Arts and Humanities, Edith Cowan University, Western Australia, Australia

**Keywords:** Ghana, parents, psychoactive substance, small-scale mining, young adults

## Abstract

Psychoactive substance misuse tends to affect the social, economic, and mental health of young adult users and their parents. However, there is currently limited research on the challenges of parents of young adults who misuse psychoactive substances. The stress-strain-coping-support theoretical model was used to assess the challenges parents of young adults who misuse psychoactive substances encounter in Ghana. Based on interpretive phenomenology, we purposively sampled and conducted in-depth interviews with 15 parents from the Adum-Banso Health Centre. Data was processed with NVivo 12 pro data and analysed thematically with four stages of Interpretative Phenomenological Analysis (IPA). The challenges of parents of young adults engaged in psychoactive substance misuse included financial constraints, the onset of negative emotional effects on parents, structural challenges in pursuit of medical treatment, and stigma and discrimination. We have provided empirical data to inform policy decisions on establishing social protection and psychosocial support schemes for young adults misusing psychoactive substances and their parents to enhance the rehabilitation of young adults recovering from the impacts of psychoactive substance misuse.

## 1. Introduction

The phenomenon of psychoactive substance misuse among young adults has become a growing global public health problem that affects individuals, families, and communities. Globally, the prevalence of psychoactive substance (e.g., alcohol, khat, tobacco, cannabis, tramadol, snuff, opioids, cocaine, and amphetamines) misuse among young adults varies across different regions and countries (Gakidou et al., 2021; Peacock et al., 2019; Peltzer et al., 2014).

In a systematic review and meta-analysis of 358 studies from 102 countries, the estimated global prevalence of substance use among young adults (15–24 years old) was 18.3% in 2019 (Gakidou et al., 2021). A study on alcohol use among young adults (15–24 years old) in 2016 reported a global prevalence of 48.8%, with the highest prevalence rates in Europe (66.2%) and the Americas (62.1%) (Peacock et al., 2019). Ogundipe et al. (2018), reveal that in sub-Saharan Africa, the overall occurrence of “any substance use” among adolescents is 41.6%, with alcohol and tobacco recording the highest prevailing substances (i.e. 40.8% and 45.6%, respectively). Additionally, the prevalence of any substance use among young adults (18–35 years old) in South Africa in the past year was 43.9%, with alcohol being the most frequently used substance (41.7%), followed by cannabis (19.7%) and methamphetamine (3.9%) (Peltzer & Pengpid, 2020). Similarly, in Ghana, the prevalence of the misuse of any substance in 2017 was 27.6%, with cannabis as the most frequently used substance (22.9%), followed by alcohol (15.4%) and tobacco (4.4%) (Owusu-Dabo et al., 2018).

The misuse of psychoactive substances in Ghana has short-term consequences (e.g., impaired judgment and coordination) and long-term effects (e.g., addiction, mental health problems, and physical health issues) (World Health Organization, [WHO] 2018). As rightly put by Taylor (2011), one of the consequences of psychoactive substance misuse by young adults could be linked to various dangerous practices, including crime, violence, and aggression. In this study, we define young adults as persons between the ages of 15 and 35 years (Ghana National Youth Policy, [GNYP] 2010). The World Health Organization (WHO, 2021) define psychoactive substances as compounds that alter mental processes such as cognition, perception, consciousness, and emotions when consumed or delivered into the body. For this study, the term “psychoactive substances” refers to cocaine, cannabis (marijuana), alcohol, tramadol, crack, and glue.

Research on parenting in traditional Ghanaian settings indicates that childbearing responsibilities were not left to biological parents alone, but the extended family even though the former is regarded as the “best caregivers” (Coe, 2014, p. 14). According to Kpoor (2015), this has witnessed a significant change as urbanisation and migration have caused the extended family support system to decline, with many paying more attention to the nuclear family.Ghana’s Western Region is known for its rich mineral resources, including gold, which has led to the establishment of numerous small-scale mining communities (Yeboah & Nyarkoh, 2022). The small-scale mining sector is a vital source of income and employment, particularly for young adults (Hilson & Osei, 2014; Nunfam et al., 2019a). However, the demand for labour in these communities has attracted young adults, many of whom engage in psychoactive substance misuse (Thorsen, 2012). Small-scale mining activities in these communities are characterised by informality and illegality coupled with the use of high-risk rudimentary methods (Nunfam et al., 2019b). Miners often misuse substances to cope with the drudgery, labour intensity and risky nature of the mining work. While indulgence in small-scale activities may be a combination of an expression of masculinity vis-a-vis having an income source (therefore disposable cash) (Nunfam et al., 2021a), there is also the issue of using substances to work for longer periods. The greater availability of substances could contribute to greater consumption (Hasin, 2018), with young adults engaging in substance misuse to cope with the physical demands of mining work coupled with heat exposure and climate change (Nunfam & Afrifa-Yamoah, 2021; Nunfam et al., 2021b), social pressures, and personal problems (Thorsen, 2012).

While the misuse of psychoactive substances among young adults in small-scale mining communities of Ghana’s Western Region is a cause for concern, little evidence based on empirical research is known about the challenges faced by parents of young adults engaged in psychoactive substance misuse. Given the problem and gap in research, a study must be conducted to accentuate the challenges encountered by parents of young adults engaged in psychoactive substance misuse in small-scale mining communities in Ghana.

## 2. Literature review and conceptual framework

### 2.1. Financial challenges

The financial effects of substance misuse among young adults on the family in small-scale communities manifest in various ways. According to Hoeck and Van Hal (2012), substance misuse places an excessive burden on the parents of substance misusers. One of the problems with psychoactive substance misuse is that it drains the physical, intellectual, and economic resources of the user and their families (Hoeck & Van Hal, 2012). Ae-Ngibise et al. (2015) argue that financial pressure is one of the significant hurdles facing parents dealing with individuals with mental health disorders. Specifically, young adults who may have been employed actively in the mining industry but for some reason stopped would find a way of acquiring possessions and other means to have money for psychoactive substances (Pieterse, 2019). Further, parents offered financial support to young adults receiving medical care, considering that young adults would take a break from work, and receive medical treatment at the hospital for over a year. This was done by parents to offset the financial burden associated with healthcare. Consequently, out of pity and sympathy, parents sold their possessions or went for loans to help treat illness associated with the misuse of psychoactive substances by their young adult children. In Ghana and Nigeria, Quinn (2007) and Igberase et al. (2012) respectively observed that parents spent so much time and money looking after their families that they barely had time for any of their social needs in African environments. In Nigeria, for example, parents classified financial issues as a greater burden than in other regions, as indicated by Ae-Ngibise et al. (2015), such as the responsibility of disturbance of family practice and collaboration, social stigma, and relative anguish. Pacheco et al. (2020) asserted that parents were more careful about going away on holiday and leaving their young adults who misuse psychoactive substances at home because they worried about what could take place in their absence or what could be missing upon their return. Ceballo and Borqueza (2018) averred that single parents experience many difficulties, and this applies mostly to women. The explanation is that even though most of their participants were women, it is also because they play a primary role in parental responsibilities, workplace expectations and other life commitments.

### 2.2. Structural challenges

In both low-income and middle-income countries in Africa, it is estimated that between 76% and 99% of people with serious mental disorders do not have access to the treatment they need for their mental health issues (Faydi et al., 2011; World Health Organization, 2009). As Ofori-Atta et al. (2010) opined, mental health is sometimes given the least priority by stakeholders in low-income countries, including Ghana. According to Sottie et al. (2016), like most African countries, Ghana has not developed adequate infrastructure and public services and systems including mental health care infrastructure to keep pace with population growth. There is inadequacy of practitioners of mental health, insufficient resources, pervasive stigma, and insufficient regional delivery of services (Ofori-Atta et al., 2010). Similarly, political apathy and stigma hinder the progress of mental health care in Ghana (Read et al., 2009). Stigmatisation of mental illness is a severe problem since it has a negative impact on patients, their families, institutions, and healthcare workers who work with people with mental illnesses (Barke et al., 2011). Stigma and prejudice make it harder for those who have or are thought to have mental disorders to obtain resources, which hinders prevention and treatment efforts and worsens the effects of mental health problems. Even though there is mounting evidence that mental health is crucial for growth, people with mental disorders, mental health services, practitioners, and even the notion of mental health itself are stigmatised and subject to discrimination around the world (Sadik et al., 2010).

The study of cultural influences on mental health is crucial for modern mental health practice, education, and advocacy. Cultural constructions of illness (Conrad & Barker, 2010) affect perception, experience, and management (Helman, 2001) of mental illness. Mental illness construction takes place at multiple levels—at the individual level, at the family level, in the community level, and in society level—and varies from lay to the professional level. In Ghana, the concept of mental illness includes an array of knowledge such as poor self-care, deficits in social functioning, disruption in behaviour, unpredictable agitation; disheveled, unkempt, or unusual appearance and disordered behaviours (Opare-Henaku & Utsey, 2017). Most Ghanaians ascribe the aetiology of mental illness to spirituality attributable to supernatural powers or evil spirits (Opare-Henaku, 2014). Consequently, society tends to distance itself from individuals with mental illness. On a more specific matter, the issue of substance misuse is not much of a difference as segments of Ghanaians attribute the excessiveness to be demonic which requires pluralistic treatment such as exorcism (Baffoe, 2013), sending patients to prayer camps or traditional and faith-based healers (Kpobi & Swartz, 2018). Some scholars (Read et al., 2009) have argued that political apathy towards mental health combined with widespread stigma, hinders the progress of mental health care in Ghana. The stigmatisation of mental illness is a serious issue given that it adversely affects patients and their relatives as well as institutions and health care personnel working with persons with mental illness (Barke et al., 2011). Across the world, people with mental disorders, mental health services, mental health professionals and even the very concept of mental health receive negative publicity and are stigmatised and discriminated against despite growing evidence of the importance of mental health for development (Sadik et al., 2010). The majority of Ghanaian mental health patients and their families consult traditional practitioners who understand them and their worldviews (Opare-Henaku, 2014). This is because, as pointed out by the authors, clinicians fail to “speak the language” of the people as they employ a lot of Western models to treat mental illness. In general, Ghanaians have a wide range of healthcare options including hospitals, traditional herbalists, and spiritualists. Previous studies in Ghana (Opare-Henaku, 2014; Roberts, 2001), and other parts of Africa (Adewuya & Makanjuola, 2008), indicate that people in sub-Saharan Africa have a preference for traditional and spiritual healers as their first line of treatment of mental illness.

### 2.3. Stigma and discriminatory challenges

Prejudice reactions impede prevention and recovery attempts and exacerbate the effects of mental health conditions (Stangl et al., 2019). Family members accept that stigma harms the relative’s self-esteem, ability to keep friends, success in getting a job or place to live, and acceptance by practitioners of mental health. (Ahmedani, 2011). The stigma imposed on persons misusing psychoactive substances extends to people with whom those persons are associated (Moses, 2014). In the United States, parents feel unwanted, despised, and rejected by their families and get a feeling that they are not considered equally important by community members (Kirst-Ashman, 2016). Such parents feel discriminated against by their community and have to overcome the difficulties of being called names because of the addiction of their young adult children to psychoactive substances. In Ghana, persons who have or are considered to have mental health issues have difficulty accessing services due to stigma and prejudice (Dako-Gyeke & Asumang, 2013). In addition, there are increased levels of anxiety, worries, feelings of extreme depression and mood swings among parents of young adults who are psychoactive substance users (Duah, 2017). According to ADFAM (2012), parents would avoid other people and conceal their relative’s situation for fear of negative reactions and stigmatisation. The World Health Organization (WHO, 2014), for example, has linked pain, disability and insecurity to the stigma and prejudice associated with mental illness (Crabb et al., 2012). Ae-Ngibise et al. (2015) on the contrary, postulate that many parents have adverse societal associations because they are not in the situation to move and interact freely with other colleagues.

### 2.4. The stress-strain-coping-support conceptual model

We employed the stress-strain-coping-support (SSCS) model as the theoretical lens to understand the challenges encountered by parents of young adults associated with psychoactive substance misuse. The SSCS model is often used to describe issues surrounding psychoactive substance misuse and the dysfunctional attributes families may experience (Velleman & Templeton, 2003). The model assumes that both the psychoactive substance users and their families face countless traumatic events as a result of substance misuse. Family members are also often plagued by such problems, which are usually long-standing, and place family members at risk of physical and/or psychological ill-health stress.

The SSCS model explains that a relative’s psychoactive substance misuse is a persistent stressor, placing strain on family members and contributing to stress-relationship. Jiang et al. (2015) note that family members are often affected by caring for a relative, especially when the relative drinks large amounts of psychoactive substances per session. The SSCS model suggests that due to the high consumption of a relative’s psychoactive substance use, the affected family members are affected by a highly stressful family environment. Various negative effects on their physical and psychological health, such as anxiety, hopelessness, depression, or fear, could be reported by the affected family members.

The SSCS model sees affected members of the family, particularly parents as average individuals trying to deal with traumatic events that are not of their own making (Orford et al., 2010). Arguably, affected family members’ ability to cope very well and have a good support system is dependent on the extent of family members’ maladjustment to the stress and strain phase (Orford et al., 2010). The SSCS model consists of four building blocks as follows: stress, strain, coping and support.

#### 2.4.1. Stress and strain

In the context of this study, stress may be defined as a feeling of physical, psychological, or emotional tension which is developed by different circumstances or events that occur in our lives (Jallow, 2020). The change that comes with the stress could cause physical, emotional, or psychological strain such as anxiety, depression, headaches, migraines, and worries to the parents of psychoactive substance users. Stress and strain assume that when a family member is addicted to either narcotics or alcohol, it is incredibly difficult for everyone who is a close family member as well as for the person whose problem is drinking or taking substances. This is because severe drinking or substance issues are associated with a variety of attributes that are harmful to intimate relationships and could be extremely difficult to live with (Adams et al., 2008; Orford et al., 2010). Adams et al. (2008) is of the view that there could be a development of a strong connection to the substance or behaviour because of the addiction, in a manner that the resources of the user such as attention, time and money are redirected away from their primary life obligations such as family, work or schooling. According to the proponents of the model, stress has variables such as parents having problematic relationships with the psychoactive substance user, time and attention being redirected from other family members to the user, family disharmony, acquiring valuable materials from parents, and parents also spending financially on the user.

A direct effect of stress is strain. In other words, when the associated difficulties become so overwhelming, it puts a burden on close families, and this leads to strain. In the context of this study, strain is a state of worry and tension caused by overwhelming difficulties at the stress level. The strain could cause physical and psychological difficulties for family members due to confusion about how to handle or cope with it. According to Orford et al. (2010), the strain is characterised by variables related to psychological symptoms such as anxiety, depression, panic attacks and physical symptoms such as headaches, hair loss, asthma, migraines, and hypertension. Consequently, the diversion of dedication to life obligations such as work and school could be traumatic for parents of the psychoactive substance user because of intense attachment to the misuse of psychoactive substances (Orford et al., 2010).

#### 2.4.2. Coping

According to Orford et al. (2010), there are three coping strategies affected family members may use. In the case of engaged coping, the family member tries to monitor and protect the family structure through assertive, supportive, or emotional reactions. Tolerant-inactive coping requires elements of approval or encouragement of the misuse of substances and facilitates the relative’s self-sacrificing actions. The withdrawal coping method applies to methods that allow family members to concentrate on their own needs and maximise the gap between them and their relative alcohol misuse (Orford et al., 2010).

The model’s core premise is that family members are faced with challenges of how to interpret and what to do about what is going wrong in the family. It involves mental difficulty and several uncertainties, in particular the main dilemma of how to respond to the relative whose conduct is an issue with drinking or substance-taking. According to Orford et al. (2010), parents usually devise strategies to deal with their young adults’ psychoactive substance misuse which are counter-productive; closely monitoring the psychoactive substance user, attempting to confine the psychoactive substance user, or regularly searching for psychoactive substance and destroying them.

#### 2.4.3. Social support

According to Orford et al. (2010), strong social support is seen as a major coping resource for affected family members. The two elements are closely connected: coping and support. Social support could be both informal and formal and is not limited to support from the closest members of the social network (Orford et al., 2010). Good social support cannot simply be equated with the number of people who exist in a family member’s close social network. It is the quality of social support that is thought to be important; and in the context of an addiction problem in the family, it is specifically a question of how well the support that a family member receives from others assists the family member in coping adequately with psychoactive substance misuse (Orford et al., 2010). According to the proponents of the model, some members would offer support in diverse ways to parents living with young adults using psychoactive substances. It could be emotional, informational, and material support. The four elements of the model are useful in this study to better understand and recognise the various variables and to gain an in-depth understanding of the experiences of young adults’ parents using psychoactive substances (Orford et al., 2019).

### 2.5. Study aims

This study aims to shed light on the unique challenges of parents of young adults who misuse psychoactive substances in small-scale mining communities in Ghana. Specifically, we analysed qualitative data from interviews with 15 parents of young adults who were receiving treatment for substance misuse at the Adum Banso Health Centre in Ghana. It is also hoped that this paper will help de-stigmatise substance misuse to help individuals seek and receive treatment at various health facilities. This study could help inform policies and the development of effective interventions aimed at supporting parents in managing the substance misuse of their young adults and promoting healthier family dynamics in Ghana and other countries facing similar challenges and ultimately contribute to the reduction of substance misuse and their negative consequences.

## 3. Materials and methods

### 3.1. Research philosophy and design

Based on the qualitative research philosophy of interpretive phenomenology, we used the descriptive research design to understand the nuances of challenges experienced by parents of young adults who misuse psychoactive substances (Bryman, 2012; Creswell & Creswell, 2017). The research design aided in understanding the challenges of parents of young persons involved in psychoactive substance misuse from the perspective of an individual and a population rather than just generalised results (Onwuegbuzie & Collins, 2007). The use of descriptive and interpretive phenomenological inquiry is to provide insight into the lived experiences of parents of young adults engaged in psychoactive substance misuse in small-scale mining communities in Ghana (Byrne, 2001; Priest, 2002).

### 3.2. Study setting

**Figure 1.**
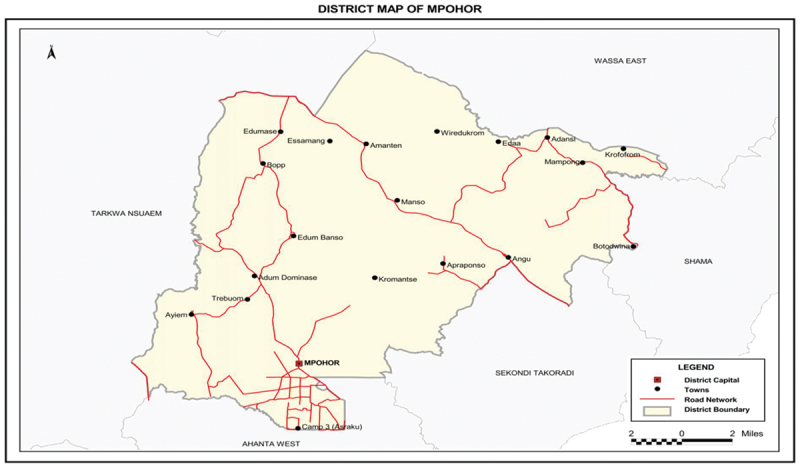
Mpohor district map.

Source: Ghana Statistical Service (2021).

The study was carried out in the Mpohor district of Ghana’s Western Region (Figure 1). The district covers a land size of 524,534 square kilometers (Ghana Statistical Service GSS, 2021) in the south-eastern part of the Western Zone. The Mpohor district has a population of 52,473 consisting of 26, 979 (51.4%) males and 25,473 (48.6%) females which represents 2.5% of the Western region’s total population (GSS, 2021). The Mpohor mining area hosts over 90 active artisanal small-scale mining (ASM) activities (Mantey et al., 2020). According to the GSS (2021), there are about nine health facilities in the district. The majority of these facilities are operated by the government, with one being operated privately. However, Adum Banso Health Centre is the only health facility in the district with a psychiatric unit to treat mental health related issues.

### 3.3. Study population, sampling procedure and sample size

The study population consisted of parents of young adults who misuse psychoactive substances such as alcohol, cocaine cannabis, crack, glue and tramadol who were being treated at the Psychiatry Unit of Adum Banso Health Centre in the Mpohor District. These six types of psychoactive substances were mostly used by young adults (Elliason, 2018; Winstock et al., 2014). Also, these combinations of substances affected their functioning in their communities. The parents of young adults who misuse psychoactive substances were considered most appropriate because of their close affinity and parental care for these young adults during the past one year at the psychiatry unit of the Adum Banso Health Centre. The treatment facility has both inpatient and outpatient status. Individuals with substance use disorders were treated at the facility. The study used a purposive sampling technique to select 15 parents of young adults engaged in psychoactive substance misuse. The lead author met with the head of the health facility detailing the purpose of the study and then with the head of the health directorate at the district capital to discuss our intentions to carry out a study in the district with her permission. The lead author reviewed folders of all patients in the psychiatric unit to confirm their level of severity and symptoms of psychoactive substance misuse. Of the patients receiving treatment at the facility, twenty-five young adults were of a higher level of misuse, hence their admission to receive care and treatment at the health facility. Higher levels of misuse of psychoactive substances by young adults in this context were those who had been affected by their social, educational, and occupational functioning as a result of psychoactive substance misuse (Hammond et al., 2014). Consequently, the total number of young adults who misused substances and had their parents available at the facility were twenty-two. Some parents had more than one child misusing substances at the time of the study. We used the opportunity to engage them about the study, and with their consent, talked about other details of the study. As a result, fifteen parents out of the twenty-two young adults present were interviewed. Two parents had already been interviewed during the pilot study, hence their exclusion from the substantive interview for the study.

The length of stay which was influenced by the severity of substance misuse was considered. Young adults who were diagnosed with substance use disorder (SUD) were considered. Also, young adults must have met at least three criteria of DSM-5 TR over a period of 12 months such as trouble stopping, effects on social and occupational functioning, physical and mental health effects, tolerance, withdrawal symptoms et cetera. Parents of young adults who were psychoactive substance users and had been at the facility less than a year were not considered for the study. At this point, as asserted by Choate (2011) parents are less likely to have rich experiences to share with the researchers. It is important to underscore that two dyads were not examined in this study.

#### 3.3.1. Data collection methods and process

We employed face-to-face in-depth interviews consisting semi-structured and open-ended questions to gather data from the participants. The adoption of face-to-face in-depth interviews enabled participants to feel at ease at expressing themselves during the data gathering process (Creswell, 2013). The structure aspects of the interview guide provided participants with the opportunity to speak specifically to issues concerning participants’ experiences. The interviews consisted of questions related to the participants’ demographic characteristics (e.g., age, marital status, number of children, number of children who misuse psychoactive substances, and number of years parents have resided in the community). The questions also specifically related to parents living with young adults who misuse psychoactive substances and the challenges they encountered in supporting the young adults.

Prior to the data collection ethical clearance was sought from the Ethics Committee for Humanities (ECH) (ECH 140/20–21), University of Ghana, in March 2021, and approved in May 2021. Also, two letters of introduction were given to the Head of the Health Centre: One from the Head of the Department of Social Work, establishing our credibility, and another from the Mpohor District Health Director, stating the need for us to be provided with the necessary assistance. Following the participants’ consent and willingness, the interviews were audiotaped with a recorder. Each interview lasted for approximately 45 minutes. The lead author conducted the interviews at a place and time appropriate to participants in Fanti or Wassa depending on the language the participants understood and could speak with ease or freely. We chose these languages because they were the major languages spoken in the district. Interviews with parents continued until saturation was attained. The lead author took field notes and kept a data collection journal to keep track of all participants and any concerns that arose throughout the interviews. Participants were appreciated with souvenir mugs worth fifty (GHS 50.00) Ghana Cedis, that is, 4.15 USD for their time and participation.

Informed Consent: Participants in the study voluntarily agreed to take part after receiving a comprehensive briefing from the lead author on their participation’s purpose, scope, and implications. Prior to the commencement of all interviews, participants provided formal consent by signing an informed consent form. For individuals unable to provide a signature, stamp pad ink was made available as an alternative means of indicating their willingness to participate. Additionally, participants were assured that all data collected would be used strictly for academic purposes, ensuring the confidentiality and ethical integrity of the research process.

#### 3.3.2. Potential harm to participants

The study could trigger emotional distress. Emotional distress refers to mental suffering as an emotional response to an experience that arises from the effect or memory of a particular event, occurrence, pattern of events or condition (Copello & Templeton, 2012). However, we provided psychosocial support arrangements for any participant who required such support. Before the start of the interview, participants were provided with the names and contact details of a trained Psychologist and a Social Worker who were available to provide psychosocial support should there be a possibility of an emotional distress.

### 3.4. Data analysis

The recorded interviews were listened to severally to acquaint ourselves with the data. The data was transcribed, and the basic themes were developed and refined in line with the research objectives. We read transcriptions and field records several times for a comprehensive understanding of participants’ experiences. Nvivo 12 pro data analysis software was used to organise and assist in the analysis of the data. We created nodes from the transcripts and generated codes based on the objectives of the study. At each stage of the analysis, we created themes and sub-themes from the data. The authors did not employ the assistance of a Research Assistant. The lead author interviewed, recorded, and transcribed the data. Coding for basic themes was also done by the lead author. Data was abridged in the form of text and quotations for easy account and analysis. We used the Interpretative Phenomenological Analysis (IPA) to analyse the data in order to give us the best opportunity to understand the innermost deliberation of the lived experiences of the research participants (Alase, 2017). Thus, the study used the four stages of Interpretative Phenomenological Analysis by Smith et al. (2021); multiple reading and making notes, transforming notes into emergent themes, seeking relationships and themes clustering, and writing up of analysis. To conceal the identities of the participants, pseudonyms were used.

## 4. Results

### 4.1. Background information of participants

The age of participants ranged from 40 to 67 years with an average age of 53.9 years. In terms of participants’ marital statuses, three were divorcees, five were widowed, three were married and four were remarried. Also, 14 participants were Christians while one was a Muslim. There were 14 females and one male. All participants were heterosexual. The age of young adults of psychoactive substance misuse ranged from 15–35 years. Table 1 illustrates participants’ background information. Table 2 illustrates the background information of young adults. All identifiable information about the participants of this present study were pseudonymised.

**Table 1.** Participant background information (*n*-15).

Participants	Gender	Age	Occupational Status Ethnicity	Educational Level
Abena	F	57	Trader Fanti	Secondary
Lydia	F	62	Trader Wassa	Secondary
Felicia	F	67	Cocoa farmer Wassa	Secondary
Selina	F	53	Artisanal farmer Wassa	Secondary
Kojo	M	52	Cocoa farmer Fanti	Secondary
Adwoa	F	45	Cash crop farmer Wassa	Basic
Ekua	F	52	Trader Nzema	Basic
Yaa	F	62	Trader Wassa	Basic
Vida	F	60	Plantation farmer Ewe	Basic
Ama	F	60	Trader Ahanta	Basic
Vera	F	55	Artisanal miner Wassa	Basic
Bernice	F	60	Cocoa farmer Wassa	Basic
Akosua	F	40	Trader Wassa	Basic
Esther	F	44	Agriculturalist Ahanta	Tertiary
Josephine	F	48	Political Party Rep Wassa	Tertiary

Source: Field Survey, 2020.

**Table 2.** Demographic characteristics of young adults.

No. of young adults in a family misusing psychoactive substance	Age of young adults (in years)	Types of psychoactive substances used	Number of siblings	Length of time misusing substances	Type of parent-dyad	Diagnosis of SUD
3 males	20 yrs	Cannabis and alcohol	7	5 yrs	Divorced	Yes
	23 yrs	Cannabis, alcohol, and cocaine	4 males	7 yrs		
						Yes
	25 yrs	Cannabis and alcohol	3females	10 yrs		Yes
1 male	32 yrs	Alcohol, cannabis,	3	12 yrs	Widowed	Yes
			1 male			
			2females			
2 males	24 yrs	Alcohol	5	3 yrs	Remarried	No
	21 yrs	Tramadol	4 males			
						No
				4 yrs		
			1 female			
1 male	25 yrs	Cocaine, alcohol, and cannabis	1 male	4 yrs	Widowed	No
1 male	22 yrs	Cannabis, crack, and alcohol	10	5 yrs	Remarried	Yes
			5 males			
			5females			
2 males	33 yrs	Alcohol, cannabis and glue	6	6 yrs	Married	Yes
			3 males			
						Yes
	35 yrs	Cannabis and alcohol		5 yrs		
			3females			
1 male	32 yrs	Crack, tramadol, cannabis, and glue	8	8 yrs	Remarried	Yes
			6 males			
			2 females			
1 male	33 years	Glue, crack, cannabis, tramadol, and cocaine	4	6 yrs	Remarried	Yes
			2 males			
			2 females			
1 male	33 yrs	Alcohol and glue	5 males	7 yrs	Married	Yes
1 male	28 yrs	Cannabis and alcohol	9	6 yrs	Married	No
			4 males			
			5females			
1 male	30 yrs	Alcohol, and cannabis	5	10 yrs	Remarried	Yes
			2 males			
			3 females			
1 male	29 yrs	Cannabis, glue, and alcohol	4 3 males	8 yrs	Divorced	Yes
			1 female			
2 males	23 yrs	Cocaine,	2 males	4 yrs	Divorced	No
	27 yrs			6 yrs		Yes
		Cannabis				
3 males	16 yrs	Cannabis and alcohol	7	4 yrs	Widowed	Yes
	18 yrs	Cocaine and cannabis	4 males			
						No
				3 yrs		
	22 yrs	Cannabis	3females			
1 male	18 yrs	Cocaine, cannabis, and alcohol	1 male	3 yrs	Widowed	No

(Number of young adults = 22).

### 4.2. Challenges of parents of young adults engaged in psychoactive substance misuse

The study yielded four sub-themes based on the experiences of parents of young adults with regards to the challenges they encounter in caring for young adults with psychoactive substance misuse. The sub-themes included financial challenges, challenges in pursuit of medical treatment, stigma and discrimination towards parents of young adults and the onset of negative emotional effects on parents.

#### 4.2.1. Sub-theme 1: financial constraints

Ten participants indicated the incidence of financial burden as a result of their young adults’ substance misuse. Even though some of the young adults were no longer productive at the mining sites, young adults could no longer afford to provide for their treatment at the health facility in addition to some of their own needs. The severity of the symptoms hindered their effective return to work at the mining sites. Parents therefore bore the cost of treatment of young adults in various ways by forfeiting their trades, rescheduling their itineraries, engaging in ancillary mining activities, requesting for financial support in the form of loans from others, and offsetting transportation and hospital bills. More specifically, single parents (divorced and widowed) emphasised on the issue of financial challenges that beset them. This might have partly been their inability to fully provide for the financial needs of their young adult children. Most of these parents had at least three children to care for. For example, being a single parent and looking for six children implied that parents defied odds, including attending to the personal and health needs of their young adult children. Attention was consequently redirected from other children to the substance user receiving care and treatment which meant that parents went extra mile in ensuring the recovery of their young adult children. Mostly, parents stayed at the regional health facilities overnights. These came with financial implications for parents. The thought of seeking medical care or being referred to regional health facilities for treatment further evoked distressing emotions. Young adults who were referred to the regional health facilities from the study site encountered several finance related challenges with respect to their sleeping arrangements, buying drugs and food for their sick young adults while at the hospital. The inaccessible roads that link regional cities became the grounds for higher transportation costs for passengers, including parents sending their young adult children regularly for medical treatment. Young adults misusing substances were noted to be aggressive and violent by sections of community members in the study site. As a result, missing items in some areas of the community were blamed on the users of substances. Parents often bore the brunt of some these and compensated for such items. Over time, it became the ordinary responsibility of the parents.

In an interview a single parent who sacrificed her schedules and resources to stay by her son who misused cocaine, alcohol, and cannabis had this to say:

I would have to sacrifice my resources to compensate for any misfortune he would encounter, and this particularly affected my finances to some extent. Whenever he would be arrested by the Police, it affected my schedules because I had to ensure he was granted bail or we resolve the issue at the community level, involving some community elders. (Madam Esther, 44 years)

A 52-year-old parent shared how her son who was severely into cannabis, crack, tramadol, and glue was admitted at the hospital affected her business. This is how she shared her story:

I spent a lot of money on transportation from here [referring to the study site] when my child was transferred to the regional hospital. To make matters worse, we spent a number of days at the hospital, and I had to come home regularly to attend to my other children which really made it extremely difficult for me. I was forced to close my shop and focus on his health because I could not reject him. (Madam Ekua, 52 years)

A 62-year-old female whose child used glue, crack, cannabis, tramadol and cocaine narrated her story this way:

My issues exacerbated when my child’s sickness became severe. I incurred debt for his treatment at the hospital, but we are not seeing any improvement. (Madam Yaa, 62 years)

A participant, 60 years, with an adult child using cannabis, glue and cocaine shared how she sacrificed resources on behalf of the son:

He spent a lot of his salary on psychoactive substances. He preferred to stay home and spend all his money before he would go the mining sites again for work. It worried me because, thinking about it, he could have used this money to do something beneficial for himself. He is mostly compelled to sell off his properties to offset debts accrued from borrowing substances. Failure to fulfil promises to the lenders, my son searches in my room for a valuable to be sold. For example, he once took my mother’s 100cedis [8.36 USD] and I had to pay for it. He has done the same thing to me and one of his siblings before. I have also warned his friends not to ever come closer to him or think of taking him to the drinking spot because I will make the police arrest them. (Madam Bernice, 60 years)

A 57-year-old participant mentioned how her finances have worsened as a result of taking care of three sons using substances. She explained it this way:

It has affected me financially in a way because, I remember when he used to work in a different town far from here and whenever he binges on alcohol, I would always have to go there on several occasions to make sure everything was fine with him. The cost of transport and other expenses to the place[referring to the residence of the young adult] has really affected my finances and business very much. (Madam Abena, 57 years)

The only male participant, 52 years with an adult child who had been diagnosed with tuberculosis and has been using marijuana, alcohol, cocaine, crack, and tramadol elaborated his experience in this manner:

I cannot ignore my child in this condition even though I expected so much from him from childhood. My schedules were impacted because of the frequent hospital visits. He was later put on medication for 6 months, but he refused to take the medications after one month and it caused a relapse. When there’s any gang activity in the neighbourhood and properties get lost, most community members come to me for payment because they claim my son would definitely be part of the gang. Even when he hasn’t taken anything, he’s blamed for that, and I had to commit financially. (Uncle Kojo, 52 years)

#### 4.2.2. Sub-theme 2: structural challenges in the pursuit of medical treatment for young adults

Nine participants indicated structural challenges in seeking healthcare for their young adult children as one of the difficulties they face. The remaining participants could not speak to this as they saw this difficulty as a necessary evil. Participants in the study site expressed the high frequency of paying hospital bills of the young adults as a worrying situation for them despite their readiness to see a significant improvement in their condition. For those being single parents, it heightened their financial situation and primary role of parental responsibilities. Coupled with the financial strain, parents provided care to the young adult children throughout the night which invariably affected their personal schedules and business prospects. Inspired by the desire to fully live and deal with a healthy young adults, parents out of desperation sought traditional treatment to supplement with western models of treating substance misuse of young adults. The frustrations to see improvement in conditions of young adults also resulted in complications for parents, which led to another financial burden. The belief that mental illness is attributable to spiritual cause encouraged parents to consult traditional healers in additional to treatment given at the health facility. Despite the delay in results, parents sometimes trusted in the efficacy of traditional treatment to “westernised” treatment which caused a shift in attention to further seek for treatment for their young adult children in the health facility.

A participant who was 60 years with an adult child using alcohol explained how she sacrificed her schedules and resources to stay by her son to seek medical treatment at a health facility. In an interview she had this to say:

I mostly readjusted my itineraries to stay close by him in his room when he’s intoxicated and feel helpless. It often results in another sickness that requires visiting the hospital and most of the bills have to be borne by me. (Madam Bernice, 60 years)

Another participant narrated her ordeal as she had to pay medical bills of her young adult. This participant, 44-year-old with a young adult using cannabis, cocaine, and alcohol had this to say:

Recently he has gone to use these substances which has caused him to be vomiting blood for five days now and I am the one taking care of him. I have paid 200 cedis[16.72 USD] for his treatment since last week. (Madam Esther, 44 years)

A participant with two male young adults using cocaine, and cannabis shared her frustrations regarding the health of her young adult son as a result of the use of the psychoactive substance:

One of my sons complained about heart pains and he was told he had a heart issue at the hospital. We [referring to herself and her son] visited the hospital more frequently this time in addition to some traditional treatment for two months until the pains subsided. The eldest son has a similar issue and it’s characterised by severe cough and oozing of blood. Fortunately for him, he was given some drugs which reduced the amount of blood flow through the mouth, and it has stopped eventually. (Madam Akosua, 40 years)

#### 4.2.3. Sub-theme 3: stigma and discrimination towards parents of young adults

While seven participants underscored certain socio-cultural attitudes that serve as a barrier to their functioning in the society, a participant shared a different view on this. This participant particularly was indifferent over the substance misuse of her son because her late husband was an individual misusing alcohol and so the son learned the behaviour and attitudes of the late father. This was quite interesting because even though the participant expressed some other challenges such as stigma and discrimination being perpetrated towards her, she anticipated the use and subsequently the misuse of substances of her young adult son. The intricate web of stigma and discrimination, ranging from pointing fingers at parents by some community members, blaming parents for the behaviour of their young adult children, significantly hindered their utmost functioning in the communities. Parents began to self-isolate and develop the “not-to-be seen syndrome”, reduced family and public engagements as their contributions appeared insignificant. In addition, parents began having lesser friends as a result of the myriads of community treatment meted out to them. Parents were then left with the option of being reactive or non-bothered about stigma and discrimination perpetrated towards them. In the extreme, some parents distanced themselves from their children and would not accept any complaints about their young adult children because of the incessant nature of it. These accounts illuminated the pressing need for targeted interventions and policy measures to address the socio-cultural barriers faced by parents in providing the essential support for medical treatment for their children.

Another participant emotionally averred how discrimination affected her cooked rice business in the community:

From the time he fell sick and got slimmer because of the weed [referring to cannabis] and cocaine, people in the community were not buying from me anymore and I had already incurred debts which led to the collapse of my business. (Madam Yaa, 62 years)

This participant averred how he was discriminated against by some members of his community:

Discrimination is another challenge I face as a parent. It happens every day and even when he has gone to use these psychoactive substances and walking around in the neighbourhood, people who know him try to let others who do not know him be aware that he is my son, and they say a lot about his situation while they associate me to it. (Uncle Kojo, 52 years)

A participant not only shared how her family members ignored the ideas and suggestions of her young adult child during meetings but echoed not being listened to and taken seriously as a result of the condition of her son:

My extended family members also commented about it. It even made him not be able to give contributions at family gatherings because his ideas were not accepted due to his situation. Mine was also not considered because I couldn’t raise my child well, let alone giving suggestions at family meetings. Elders from the neighbourhood even said he is very intelligent, but his habit of drinking has not helped him achieve anything so far. (Madam Abena, 57 years)

A female participant explained how she became a subject of mockery and labelling in the community:

As for discrimination, it happens a lot and it is not easy going through such a thing. Anytime I go out, people from the neighbourhood point hands at me that this is the mother of so-and-so, the psychoactive substance users and it really disturbs me. They sometimes even had quarrels with people who label them as substance users. These people suspect my child and his friends to be stealing from them. Such issues affects me as a mother. It got to a time one of them was cut with a knife following a fight and it required visiting the hospital until he got better. (Madam Akosua, 40 years)

This is how another participant shared her discrimination ordeal from some of the community members:

Some community members here discriminate a lot because sometimes when I am walking alone in town, I hear whisperings, “that’s the mother of the guy who uses marijuana a lot” and I most often pretend as if I don’t hear what was said. After all, a parent who is quick-tempered would be forced to retaliate or take action which might result in another problem. (Madam Vera, 55 years)

A participant, 62 years old mentioned how some people in the community pointed at her that she has a son who is into the misuse of psychoactive substances:

I am a peaceful person and I do not like trouble so when I go out, people point at me and they will be saying “that is the mother of the boy who used substances till his death”. Others also say he never listened to advice and that he never pitied his poor mother which sometimes makes me shy when I am among people. they tend to talk about the use of psychoactive substances, and I pretend as if I do not know about that, and I do not even have a son who is into that. (Madam Yaa, 62 years)

However, this participant shared a contrary experience of discrimination. She recounted not being subjected to any form of discrimination in her community but felt very depressed and ashamed personally when in public with her young adult child.

I have never been discriminated because of my son’s condition because I know that is his behaviour. Besides, my late husband was in the same condition, so it was not anything new to me. However, when there is a funeral and I see my son coming around to dance, I feel very ashamed and depressed. (Madam Lydia, 62 years)

#### 4.2.4. Sub-theme 4: the onset of negative emotional effects on parents

While one participant did not explicitly respond to this, the remaining participants indicated the extent to which their experiences of emotional problems unfolded. Parents were compelled to share the same bedroom with young adults to monitor their movements and offer informational support. These were done to curtail the menace of emotional challenges that confronted the parents. The stark reality of not being in control of situation meant parents felt like they were not doing enough in the upbringing of their children. Some community members chose to insult them for no reason. Sudden mention of their names when their children might have done something unhealthy and surprising exuded mental health challenges. Parents were worried, unhappy, and uncomfortable and felt disgraced. Parents shed tears when it was realised things were out of control and felt seemingly not doing enough to protect their children. In addition, thinking about the state of young adults exerted some level of guilt. Community members having the perspective about parents’ inability to train their children well who misuse substances resulted in desperation and frustration for parents. Parents expressed how it affected their family reputation and that caused a lot of worry, anxiety, and uncertainties for them. A distinct pattern emerged on the diverse efforts by parents to be in control of the situation.

This 57-year-old participant recounted why she had to force her adult son to come to sleep in her room whenever she becomes emotionally affected:

I face a lot of challenges and it makes me cry so much when I think about it. I sometimes even have to make him come to sleep in my room overnight, so I can get the opportunity to advise him. Because going through this situation for my son has not been easy for me at all. (Madam Abena, 57 years)

This participant, 60 years old, mentioned how her son talked any time he gets intoxicated and that has made her feel very unhappy:

Yes, I go through some challenges because when he gets drunk and comes home, he talks anyhow to all of us in the home. I feel uncomfortable and unhappy about it. I feel disgraced because of how he has fully immersed himself into this habit. To the extent of collapsing at the drinking spot as a result of too much alcohol, his friends would be laughing at him while we put in every effort to ensure he is in a good state. I know it is not a good thing, so it makes me feel bad about it. (Madam Ama, 60 years)

This 67-year-old female echoed how the embarrassment she faces even extends to demeaning their family image in the community:

I get embarrassed because it affects our family image as well. He was beaten to have taken Somone’s gold. I cry mostly because of such complaints. He becomes aggressive towards me if I fail to give him food when he is intoxicated. He even threatened to kill me the other time I refused to give him money. As a result of this, I lived in fear. Also, whenever I buy shirts for him, in no time, he makes it very dirty. I feel shy when he approaches me in public. The reason is that he doesn’t eat quite well and one of his sisters usually cry when she comes to this town to visit and sees him. (Madam Lydia, 62 years)

A participant echoed how her mental health challenges manifested:

When I met them at the ghetto [referring to the temporary structures under which young adults meet and use substances], our conversation became an argument which served as a mockery for their friends towards us and they usually passed the comment “this is their time and that mine has already passed”. It makes me angry, but what I also observed was that it’s not their own will to be engaged in such acts or behaviours. So, I went there another day to see for myself the kind of substances they use, and it disturbed me a lot. After the incident, they left home from our previous place of residence to this town. They kept saying I was the cause of their behaviour and that I was always disgracing them. (Madam Akosua, 40 years)

Another participant narrated how she has been diagnosed as being hypertensive as a result of the many troubles her young adult child indulges in:

Hmm! my brother [referring to the researcher], right now as we speak, I am going through some heart problems. Because most often when I am asleep, someone would come and awaken me to come and see my son getting himself into trouble. I would usually wake up abruptly which affects the rate at which my heart beats. So recently I went to the hospital, and I was diagnosed with heart complications. Most of the troubles were about fighting. He and his siblings always fought, including fighting against their colleagues around here [referring to the neighbourhood]. (Madam (Madam Vera, 55 years)

## 5. Discussion

Following our empirical assessment of the challenges experienced by parents of young adults using psychoactive substances in the context of the SSCS theory, the study’s findings showed that these parents experienced financial constraints, challenges in pursuit of medical treatment, stigma and discrimination towards parents of young adults and the onset of negative emotions on parents.

### 5.1. Financial constraints

Firstly, parents valued their young adults’ health and safety, often committing financially to provide them with medical assistance. This added stress, especially when other children needed care. Some resorted to loans, expecting improved conditions to repay debts and support young adults’ needs. Financial strain on parents leads to increased concentrations of unmet needs. Also, changes in parental schedules to satisfy young adults’ needs might result in business losses, potentially accruing debts, especially for single parents. To this, Ceballo and Borqueza (2018) agreed that single parents experience difficulties in balancing primary parental responsibilities with work and other life commitments. The authors further added that it affected parents’ financial safety net. Parents indicated financial challenges when their young adult children were referred to the regional hospital, leading to difficulties in providing accommodation and basic needs. Parents also faced financial strain when resolving crime-related issues or addressing resources misappropriation by their young adults at distant mining sites. Furthermore, the issue of providing resources by parents to resolve conflicts that involve young adults due to psychoactive substance misuse often leads to feelings of tension and pressure (Hoeck & Van Hal, 2012). Similarly, Adams et al. (2008) noted that parents of substance users face tremendous stress as they adjust to their new reality, which could contribute to financial strain. This strain is further exacerbated when young adults exchange the belongings of their parents for psychoactive substances (Masombuka, 2013). Despite the financial challenges, parents displayed an admirable commitment to helping their children, primarily through informal means. Similar to previous research (Ae-Ngibise et al., 2015), this study demonstrated that parents provided the most support in the form of emotional, informational, financial, and material support.

### 5.2. Challenges in the pursuit of medical treatment

Transportation costs to commute from one community to another and other mining sites to seek care were challenging, thereby impacting healthcare access for those with chronic illnesses in the study site. Previous research (Syed et al., 2013) underscores transportation as a crucial role in healthcare access, especially for low-income individuals. Despite being faced with the issue of long travel time to visit their children in tertiary referral hospitals in the urban areas, parents readjusted their schedules to attend to the health needs of the young adults. Parents would have to spend a lot of time at health facilities for treatment for young adults as a result of psychoactive substance misuse. It was for this reason that Bawadi et al. (2022), highlighted challenges related to seeking medical treatment as a crucial healthcare challenge for parents of young adults using psychoactive substances. Syed et al. (2013) also confirmed transportation barriers including long-distance travels to access mental health services as barriers to mental health services.

The financial impact of the long travels in addition to the huge hospital bills compelled parents to supplement the treatment of young adults in substance misuse at the health centre with traditional care. Faydi et al. (2011) estimated that between 76% and 99% of people in both low-and middle-income countries in Africa with serious mental disorders do not have access to the treatment they need for their mental health issues. Unlike a study (Adewuya & Makanjuola, 2008) that found sub-Saharan Africans to prefer traditional and spiritual healers as their first line of treatment of mental illness, Opare-Henaku (2014) further argued that parents resorted to traditional practitioners because they “spoke the language” of the patient. The study also supports a study by Opare-Henaku (2014) that Ghanaians have a variety of healthcare options including hospitals, traditional herbalists, and spiritualists. However, Ofori-Atta et al. (2010) indicated in a study that in Ghana, there is a shortfall in the provision of mental health services due to inadequate practitioners in mental health, insufficient resources, and poor regional delivery of services. Therefore, young adults were exposed to the development of Substance Use Disorder (SUD) and Tuberculosis (TB) due to psychoactive substance misuse. We found in this study that the continuous referral of young adults to regional and metropolitan hospitals may be attributed to the shortfall of mental health staff in the health facility. We also noted that parents however first reported to the treatment facilities before augmenting with traditional methods. This section illuminates the extent to which parents were readily available to offer support to their young adult children misusing substances. This corroborates the element of support as found in the stress-strain coping support model.

### 5.3. Stigma and discrimination towards parents of young adults

Stigma and discrimination were found to be another challenge that beset parents of young adults engaged in substance misuse. Parents experienced stigma and prejudice at work, neighborhoods, and in their families. Jackson et al. (2007) highlighted the confirmation of suspicion, coping with guilt, shame and preference for self-preservation as some of the challenges parents face when young adults begin to misuse psychoactive substances within the family. Parents were stigmatised primarily because of the misuse of substances of their young adult children. Similarly, contributions by young adults were disregarded because there were seen as insignificant. For this reason, Kirst-Ashman (2016) observed that parents feel unwanted, unloved, and rejected by their families and get a feeling that they are not treated equally. The unequal treatment is usually accompanied by increased levels of anxiety, worries, feelings of extreme depression and mood swings among parents. Further, the exhibition of the psychological symptoms such as anxiety, depression and emotions reflect the display of the stress as the framework of the SSCS model illustrates (Orford et al., 2010). The type of psychoactive substances used by young adults were effortlessly linked to parents’ reputation in society. For example, some participants mentioned how some community members spoke ill about them because of the misuse of substances by their young adult children. On the contrary, one participant whose husband used substances before his demise was indifferent to the issue of stigma and discrimination. It was a principle of “like father like son” so her son’s misuse of substances meant his desire to learn from his father. However, parents felt embarrassed as their young adult children continued to misuse psychoactive substances and some community members started to talk about it.

### 5.4. The onset of negative emotions on parents

Parents were faced with challenges with stress and other emotional effects as a result of their child’s substance misuse. A disagreement mostly occurred between young adults and their parents. It also could happen outside the immediate family unit. This mostly resulted in family and community conflicts. Parents usually intervened for recurring police related cases to be resolved at the community level. Parents were devastated to notice their aggressive young adults not adhering to their advices and doing things that tainted the family’s image. Svensson et al. (2020) affirmed in their study of an individual on substance being erratic, aggressive, and destructive. Similarly, the problematic relationship between parents and young adults sheds much light on the strain element of the SSCS model as parents were initially unable to tame the challenges until it started causing cracks in the family because it was difficult to live with. Some married parents recounted the series of trauma it posed to them due to the unwillingness of the young adults to comply with their instructions at home. Parents found young adults who argued with them in front of their peers humiliating. Mathibela and Skhosana (2019) also confirmed parents’ experiences of strain as they become susceptible to negative emotions such as depression, anxiety, shame sorrow, and uncertainty. This affected the emotional state of parents because it was disgraceful for their child(ren) to argue with them in public. Parents indicated psychological defects as violence in the community by others reminded them of their young adults’ misuse of substances. The knowledge that family image could not be restored even after the young adults had stopped misusing psychoactive substances worried parents. Parents became worried and depressed and would grieve after their efforts went futile. Similarly, Smith and Estefan (2014) observed that parents were overly depressed and had a feeling of anguish.

### 5.5. The SSCS model and its implications for the study

The findings of the study shed light on the engaged coping style. Parents tried to stop or reduce young adults’ substance misuse and attempted to control their behaviour, being assertive and confronting issues whilst trying to support them. For example, a female participant indicated how she shared the same bedroom with her adult child to prevent him from further intake of alcohol and misuse of substances. This was done by the parents to offer informational support in the form of advice to the young adult misusing substances. This mostly caused some level of stress due to increased conflict in the parent-child relationship. On the contrary, a participant whose husband used substances and had passed on before the study was conducted was indifferent about the engaged coping style. She rather endorsed the tolerant (inactive) coping style. She accepted her son’s behaviour even though she was not happy about it. Parents offered financial resources and other responsibilities in the face of substance misuse of their young adult children. This indicates the engaged (supportive) coping style of parents in the study. Parents did not rely on the withdrawal coping style as they needed to show compassion, concern, and support because they could not reject their young adult children in that state at that point in their lives.

Cook (2011) posited that considering youth with psychoactive substance dependency without treating the family “limits our vision and decreases the potential for the recovery of a young life”. Pacheco et al. (2020) argues that there is reluctance on the part of parents living with young adults using psychoactive substances to ask for support due to firmly thought values about what it meant to be a good parent and the guilt that a family member would feel if it was known outside the family. Choate (2015) on the other hand, asserted that the guilt and culpability of parents also stopped them from receiving help from friends and members of the extended family. Nonetheless, according to Choate (2015), parents could rely on the family of a psychoactive substance misuser in their close circles who may be responding to treatment for psychoactive substance misuse when the problem appears to worsen. In a qualitative study by Ae-Ngibise et al. (2015), it was reported that parents of young adults who use psychoactive substances had no support. According to the authors, the intervention was for the users, but not their parents. In the study, parents ascribed the non-existent support systems to poverty; nobody, therefore, had surplus money or resources to offer. Again, the authors stated discrimination and lack of empathy as the influences responsible for the lack of support for parents.

Zimić and Jukić (2012) highlighted the profound impact of substance misuse on families, stressing that the dynamics within the family setting are significantly altered when at least one member engages in substance misuse. The study found that parents living with young adults were faced with role changes, fear, violence, disruption of routines, and social life, and negative impacts on their finances. As a result, parents were more likely to be vulnerable to stress and more susceptible to having problems with trauma, anxiety, depression, and other mental illness. Lander et al. (2013) maintained that family members are affected by the individual’s substance misuse; however, each individual is affected differently together with, but not limited to, having unmet developmental needs, impaired attachment, economic hardship, legal problems, emotional distress and sometimes violence being perpetrated. How a family copes or manages substance misuse has a profound effect on the way others experience the problem, as well as the course and severity of the problem. The family which remains the primary source of attachment, nurturing, and socialisation for humans in our current society is affected uniquely by the individual using substances including but not limited to economic hardship, emotional distress, and sometimes perpetration of violence towards other family members (Lander et al., 2013). According to Velleman and Templeton (2007), as the course of substance misuse progresses, the emotional responses of family members are worsened in families where substance misuse is prevalent. Families tend to experience considerable stress-related difficulties including insomnia, anxiety, and depression. Orford et al. (2010) further contend that isolation and suicidal ideation, betrayal and resentment from family members are present. Substance misuse is regarded as an illness of the entire family, not just the substance abuser but the other family members as well. Thus, substance misuse is viewed as a “family disease” which affects most if not all family members (Klostermann & O’Farrell, 2013). In a substance-affected family, functional family roles are often missing or distorted (Gruber & Taylor, 2006).

### 5.6. Limitations of the study

The significant contributions of this study are apparent in its use of the stress-strain-coping-support theoretical model to understand and highlight parents’ challenging experiences of caring for their young adult misusing psychoactive substances. It also accentuates empirical data which could inform mental health practitioners and policy makers on the need for psychosocial protection and coping mechanism for rehabilitating young adult misusing psychoactive substances to assuage the challenges of parents. However, this study was associated with the following limitations. This study primarily focused on the challenges of parents of young adults into psychoactive substance misuse other than the young adults and other stakeholders. Hence, future research could incorporate the perspectives of young adults and other stakeholders (e.g., psychiatric nurses, physician assistants, nurses, and psychologists) to widen the scope and perspectives of psychoactive substance misuse and its challenges. In terms of its methodology and study setting, future studies could adopt quantitative approach and data in research settings beyond the Mpohor district which could provide quantitative and wider scope in perspective of the phenomenon of substance misuse. This would help to generalise the findings of the study to the national populations to better guide policy formulations. Participants were recruited from an inpatient/outpatient psychiatric facility which could affect the study outcome because participants and young adults might feel compelled to provide information. Additional studies could look at psychoactive substance misuse from other sources, including conducting interviews outside a facility. Out of the fifteen participants, one was a male. As with this study, female participants being a larger proportion of the study sample exuded a lot of detailed exposition for this study. However, future research could consider the challenges of male parents as their experiences may differ from female parents. Since most of the female participants emerged from single-parent households, the study findings may have resulted from single parents’ difficulties dealing with young adults’ psychoactive substance misuse. The study findings may have been richer if participants were interviewed in pairs—thus, dual-parent households. With this, within the same household, divergent views could have emerged from parents in dual households. Interviews from dual-parent households on the challenges they face as a result of their child’s psychoactive substance misuse may have yielded unique study results.

## 6. Conclusions and implications

Psychoactive substance misuse by young adults poses a lot of risks and harm not only to the users but their families. Parents of young adults using psychoactive substances are beset with enormous challenges such as financial difficulties, shame and discrimination, structural challenges in seeking medical care, and mental health challenges of parents. The experiences of parents of young adults misusing psychoactive substances have implications for mental health practices, development, and policy decisions. We have provided evidence-based and empirically supported data on the challenges of parents with young adults who misuse psychoactive substances to inform mental health practices across stakeholders (e.g., psychiatrists, mental health nurses and social workers). It can also inform policy decisions on establishing sustainable social protection and psychosocial coping and support schemes for rehabilitating young adults using psychoactive. In addition, the study recommends that the Department of Social Welfare (DSW) of the district should help establish a formal support group for parents of young adults due to the misuse of substances of their children. This would create the atmosphere for parents to discuss or share their experiences to be positioned to learn from such avenues. In addition, the government of Ghana through the various District Assemblies should also consider developing a psycho-social support scheme to young adults in the Mpohor district aimed at helping them go through rehabilitation.

Further, for mental health practitioners at treatment facilities to better speak the language of the patients, it may be helpful for traditional practices to be practiced alongside the various interventions that may be offered at mental health facilities to better meet patients’ (and their families) values and preferences. This integration could be adopted if mental health practitioners are trained to better develop the requisite skills and knowledge for effective implementation at the various mental health facilities.

## Data Availability

The data that support the findings of the study are available from the lead author, upon reasonable request.

## Appendix I

### Demographic information

1. What is your age————–?
2. How many children do you have————?
3. How many of them use psychoactive substances? —————-
4. What is the age of your ward (s) using psychoactive————-?
5. What is your religion?

Christian————Muslim——————

Traditional religion———-Other————Please specify—————

6. What is your marital status?

Married———Divorced————–Single————–

Cohabiting———–Widowed——————Separated—————–

7. For how long have you been in this community?

1 year————-2 years ————-3 years—————-

4 years———–Other—————Please specify—————

## Appendix II

### Interview protocol for parents living with young adults

I am [name withheld], and I would like to introduce myself to you. I’m a social work student at the [name of University withheld]. As part of my MPhil program, I’m conducting research on psychoactive substance use among young adults in small-scale mining communities: experiences of parents in the Mpohor District of the Western Region.

Your input will help me develop a greater understanding of the perspectives of parents of young adults who use psychoactive substances such as marijuana, tramadol, tobacco and alcohol. A set of questions will be posed to you. This interview is for academic purposes only, and all information given will be kept private.

#### A. Factors contributing to psychoactive substance use by young adults

1. Do you have any of your wards using psychoactive substances?
2. What kind of psychoactive substances do your ward use?

Alcohol ———–Marijuana ————Tobacco —————

Tramadol ————Inhalant ————–

3. Could you please tell me about how you found out that your ward was on psychoactive substances?
4. What contributed to your ward’s psychoactive substance use in this area? Probe (has your son been involved in galamsey activities? If yes, for how long? Will you say galamsey has also contributed to this?).

#### B. To understand the problems parents of young adults who use psychoactive substances face

5. Do you go through any challenges by virtue of your ward using psychoactive substances?
6. What are some of the challenges you have encountered as a result of your ward using psychoactive substances?
Probe (family, stigma, discrimination, depression, finances, work-related)
7. Have you had any experiences where the other siblings have indicated you are using so much resources on the psychoactive substance user?

#### C. Strategies parents use to cope with young adults who use psychoactive substances

8. How do you cope with your ward using psychoactive substances?
Probe (Individuals, family, support groups, friends, neighbours, community members, hospital, police, individuals).
9. In what ways do they provide support?
Probe (Financial, health care, emotional, psychological, instrumental, care arrangements).
