# Correction

**DOI:** 10.1080/28324765.2025.2457874

**Published:** 2025-01-29

**Article title**: Challenges of parents of young adults misusing psychoactive substances in small-scale mining communities in Ghana

**Authors**: Philip Asamoah, Samuel Nana Abokyi and Victor Fannam Nunfam

**Journal**: Cogent Mental Health

**DOI**: 10.1080/28324765.2024.2382182

This article was originally published with an error in the text. To rectify this, the following additional content has now been included at the end of the ‘Data collection methods and process’ section.

Informed Consent: Participants in the study voluntarily agreed to take part after receiving a comprehensive briefing from the lead author on their participation’s purpose, scope, and implications. Prior to the commencement of all interviews, participants provided formal consent by signing an informed consent form. For individuals unable to provide a signature, stamp pad ink was made available as an alternative means of indicating their willingness to participate. Additionally, participants were assured that all data collected would be used strictly for academic purposes, ensuring the confidentiality and ethical integrity of the research process.

